# Mesenchymal stem cell transplantation alleviates experimental Sjögren's syndrome through IFN-β/IL-27 signaling axis

**DOI:** 10.7150/thno.37351

**Published:** 2019-10-21

**Authors:** Genhong Yao, Jingjing Qi, Jun Liang, Bingyu Shi, Weiwei Chen, Wenchao Li, Xiaojun Tang, Dandan Wang, Liwei Lu, Wanjun Chen, Songtao Shi, Yayi Hou, Lingyun Sun

**Affiliations:** 1 Department of Rheumatology and Immunology, The Affiliated Drum Tower Hospital of Nanjing University Medical School, Nanjing, China.; 2 The State Key Laboratory of Pharmaceutical Biotechnology, Division of Immunology, Medical School, Nanjing University, and Jiangsu Key Laboratory of Molecular Medicine, Nanjing, China.; 3 Department of Pathology and Center of Infection and Immunology, University of Hong Kong, Hong Kong, China; 4 Mucosal Immunology Section, NIDCR, NIH, Bethesda, Maryland, USA; 5 Department of Anatomy and Cell Biology, University of Pennsylvania, School of Dental Medicine, Philadelphia, USA.

**Keywords:** Mesenchymal stem cells, Sjögren's syndrome, IL-27

## Abstract

**Rationale**: Although mesenchymal stem cell (MSC) transplantation has been proved to be an effective therapeutic approach to treat experimental Sjögren's syndrome (SS), the detailed underlying mechanisms remains unknown. IL-27 has diverse influences on the regulation of T cell differentiation and was involved in SS through modulating immune response. Here we aimed to explore whether IL-27-mediated regulation of immune cells was responsible for the beneficial effects of MSC transplantation on SS.

**Methods**: The SS-like symptoms were evaluated in IL-27 deficient and recombinant IL-27-treated NOD mice. The MSCs were infused into NOD mice via the tail vein. The histological features of submandibular glands, saliva flow rate and serum IL-27 were examined. The effects of MSCs on the IL-27 production and Th17/Treg cell in SS patients and mice *in vitro* and *in vivo* were determined for the mechanistic study.

**Results**: This study showed that SS patients had decreased IL-27 level and increased ratio of Th17/Treg cells. Consistently, exacerbated SS-like symptoms were observed in IL-27 deficient NOD mice, along with increased ratio of Th17/Treg cells. Importantly, MSC transplantation alleviated SS-like symptoms by elevating the level of IL-27 to restore Th17/Treg balance in NOD mice. Mechanistically, MSC-secreted interferon-β (IFN-β) promote dendritic cells to produce IL-27.

**Conclusions**: Thus, we have revealed a previously unrecognized function of MSC-mediated IL-27 production by DCs in suppressing SS-like syndrome, which provided evidences for clinical application of MSC in patients with SS.

## Introduction

Primary Sjögren's syndrome (SS) is a systemic autoimmune disorder characterized by lymphocytic infiltration of salivary and lachrymal gland leading to dry mouth and eye dryness progressively. There are many systemic features of SS, such as fatigue, Raynaud's syndrome, dry skin, joint and muscular pain 1-3. Current therapies are mainly focused on relieving the symptoms with no approach to cure SS.

A promising therapeutic option for SS is MSC transplantation. MSCs are multipotent stem cells present in many adult and fetal tissues, such as bone marrow, adipose tissue, liver, dental pulp, umbilical cord blood, and placenta. MSCs have the capacity to differentiate into several cell types from the mesodermal germ layer, such as adipocytes, osteocytes, and chondrocytes 4-6. Due to their low-immunogenicity, safety, efficiency, abundance and easy collection, MSCs have been used to treat various human diseases 7-10. The preliminary animal and human studies, including ours, showed that MSC transplantation could reestablish salivary function and reduce lymphocytic infiltration in salivary glands 11,12. However, the mechanisms of the encouraging results of MSCs on the SS remain to be elucidated.

It is known that T cells are in the center of pathogenesis of some autoimmune diseases. Studies have indicated that the balance of regulatory T (Treg) cells and T helper 17 (Th17) cells is a key factor in maintaining immune homeostasis. Generally, the abnormal activation of Th17 cells promotes inflammation and induces autoimmune reactivity, while Treg cells inhibit inflammatory responses 13,14. Consequently, Th17/Treg imbalance may participate in the pathogenesis of autoimmune diseases, including primary Sjögren's syndrome. However, the underlying mechanisms leading to the imbalance between Treg and Th17 cell commitment in SS are not fully understood.

It is known that many cytokines are involved in maintaining the balance between Treg and Th17 cells. Interleukin-27 (IL-27) is a member of the IL-12 family. The IL-27 receptors (IL-27R) are expressed by a variety of hematopoietic and immune cells. Previous studies showed that IL-27 and IL-27R interaction plays an important role in immune function. Naïve CD4^+^ T cells express a low level of IL-27R, indicating that IL-27 regulates CD4^+^ T differentiation. IL-27 promotes the differentiation of Th1 cells, but inhibits the differentiation of Th2, Th17 and Treg cells 15-17. IL-27 is mainly produced by activated antigen presenting cells, including dendritic cells (DCs) 18. Accumulating evidences have demonstrated that MSC transplantation regulates the function of DCs 19,20. Therefore, we aimed to explore whether IL-27-mediated regulation of Th17 and Treg cells was responsible for the therapeutic effects of MSC transplantation on SS.

## Materials and methods

### Patients and healthy controls

All SS patients in the present study, who were hospitalized at the clinical unit of rheumatology and immunology of the Affiliated Drum Tower Hospital of Nanjing University Medical School from January 2014 to December 2016, were recruited. The patients were diagnosed according to the 2002 American-European Consensus Group criteria for SS 21. The detailed inclusion and exclusion criteria were described in our previous study 12. The demographic and treatment data as well as duration and disease activity of SS patients are summarized in the **Supplemental Table 1**. The age- and sex-matched healthy volunteers served as control. The study follows the ethics guidelines of our hospital and registered at www.clinicaltrials.gov (NCT00953485). Written informed consent was obtained from each patient and healthy volunteer. All research with human subjects was in compliance with Helsinki Declaration.

### Mice

The non-obese diabetic (NOD) mouse is the widely used model of SS. The NOD mice develop histopathological changes in the salivary glands and autoantibodies in serum, as those patients with SS do 22. In this research, NOD/Ltj mice were purchased from the Model Animal Research Center of Nanjing University. The IL-27^-/-^ NOD mice were generated by the lab of the Model Animal Research Center of Nanjing University. According to previous study, ICR (Institute of Cancer Research) mice, which the NOD strain was derived from, served as control 12. All the mice used in this study were housed in a normal 12h/12h light/dark cycle. Mice had access to diet and water *ad libitum*. Saliva flow rate of each mouse was determined as described previously 12. In separate experiment, 11-week-old IL-27^-/-^NOD mice were injected intraperitoneally with murine recombinant IL-27 (300 μL, 200 ng/mL per mice) for 7 consecutive days or equal volume of PBS.

### Culture and transplantation of MSCs

The umbilical cord-derived MSCs (UC-MSCs) were from the Stem Cell Center of Jiangsu Province. The detailed purification and identification procedures were described previously 12,23. The UC-MSCs were cultured in Dulbecco's modified Eagle's medium with low glucose containing 10% fetal bovine serum. For SS patient treatment, UC-MSCs at passage 4 (1 ×10^6^/kg of body weight) were administered by intravenous infusion without premedication, such as steroids or antihistamines. For animal experiment, the MSCs at passage 4 were suspended in PBS and infused into NOD mice (1×10^6^ cells/mouse) via the tail vein at 8 weeks of age. After 4 weeks, the mice were sacrificed and the tissues were collected for analysis.

### Culture of human and mouse dendritic cells

Human monocyte-derived DCs were generated from CD14+ cells according to previous study 24. Briefly, peripheral blood mononuclear cells (PBMCs) were isolated from buffy coats of SS patients and healthy controls by lymphoprep^TM^. CD14+ monocytes were purified by microbeads (MiltenyiBiotec). The CD14+ monocytes were induced to DCs with the presence of IL-4 and granulocyte macrophage colony-stimulating factor for 6 days. For detection of IL-27 expression of monocyte-derived DCs, monocyte-derived DCs from SS patients and healthy controls were generated and cultured in complete RPMI 1640 with 100 ng/mL LPS.

Mouse DCs were purified according to the previously described protocol 25. The spleen was dissected from mice and gently grinded. The cells were passed through a mash and the erythrocytes were lysed. Cells were resuspended in 2mM EDTA PBS containing 0.5% heat-inactivated fetal calf serum. Then the cells were mixed with CD11c antibody labeled microbeads (MiltenyiBiotec) at 4℃ for 15min. The CD11c-positive cells were collected and resuspended in RPMI1640 media. To determine the expression of IL-27 by mouse DCs, 100 ng/mL LPS was added in media to induce the activation of DCs.

### Coculture of MSCs and monocyte-derived DCs

To evaluate the effects of MSCs on the IL-27 production by monocyte-derived DCs, a trans-well system was used to perform the co-culture experiment. MSCs were seeded into the trans-well membrane of the inner chamber with 0.4μm pore size. The monocyte-derived DCs were plated in the lower chamber. The ratio of MSCs to monocyte-derived DCs was 1:10. The cells were cultured in complete RPMI 1640.

### Histological examination

The labial glands of SS patients were collected and fixed in 4% paraformalclehyde overnight, processed and embedded in paraffin. The tissue sections were stained with hematoxylin and eosin (H&E). The submandibular glands of mice were also collected for histologic analysis.

### Real-time polymerase chain reaction

The RNAs were extracted from whole blood by Trizol method (RNAiso blood from Takara). cDNA was synthesized by the SuperScript III First Strand Synthesis SuperMix. The PCR reaction was run on the StepOnePlus^TM^ Real Time PCR Systems and analyzed by StepOne Software. The primers were listed in **Supplemental Table 2**. The relative gene quantification was done using the 2^-△△^Ct method following normalization to glyceraldehydes-3-phosphate dehydrogenase.

### Flow cytometry

PBMCs were purified from peripheral blood of SS patients by Ficoll density-gradient centrifugation. Then PBMCs were incubated with anti-human antibodies to characterize cell subsets: Th17 cells (FITC-CD4, PE-IL-17A) and Treg cells (FITC-CD4, APC-CD25, PE-Foxp3) respectively. For mice, a single-cell suspension was collected by mincing the spleen between the frosted ends of glass slides. Then the cells were stained with following anti-mouse antibodies: FITC-CD4 and IL-17A for Th17 cells. For Treg cells, T cells were stained with FITC-anti-CD4, APC-anti-CD25 for 30 min on ice. Then, cells were stained with anti-Foxp3 antibody using Foxp3 staining buffer kit from eBioscience. The stained cells were assayed by a FACSCalibur flow cytometer and data were analyzed with FlowJo software.

### ELISA assays

To evaluate protein levels of IL-27, TGF-β and IL-17A in serum and cell culture supernatants, ELISA assays were performed by ELISA kits from R&D Systems according to the manufacturer's instruction. Human and mouse IgG, IgM and IgA of serum were also determined by ELISA kits from Invitrogen and Bethyl Laboratories.

### siRNA silencing of IFN-β in MSCs

The expression of IFN-β by UCMSCs was down-regulated by small interfering RNA technique. The small interfering RNA (siRNA) and negative control (non-target control) were designed and synthesized by Biomics Biotechnologies Company (Nantong, China). The IFN-β siRNA sequence: GCUGGAAUGAGACUAUUGUdTdT---ACAAUAGUCUCAUUCCAGCdTdT. The negative control sequence: UUCUCCGAACGUGUCACGUdTdT—ACGUGACACGUUCGGAGAAdTdT. The siRNA was transfected into MSCs with Lipofectamine 2000 according to the instruction of commercial kit from Invitrogen. The effects of interference were assessed by real-time PCR.

### Statistical analysis

Quantitative data were presented as mean±SEM. All statistical calculation was performed with GraphPad Prism software. For the normally distributed data, the significance was analyzed by Student's t tests for comparison of two groups or by one-way ANOVA followed by Bonferroni's test for multiple groups. For the non-normally distributed data, the Mann-Whitney U test or the Kruskal comparison test was applied. P value less than 0.05 was considered significantly different. Multiple group comparison was performed by one-way ANOVA.

## Results

### The level of IL-27 negatively correlates with disease activity in SS patients

**Figure 1:**
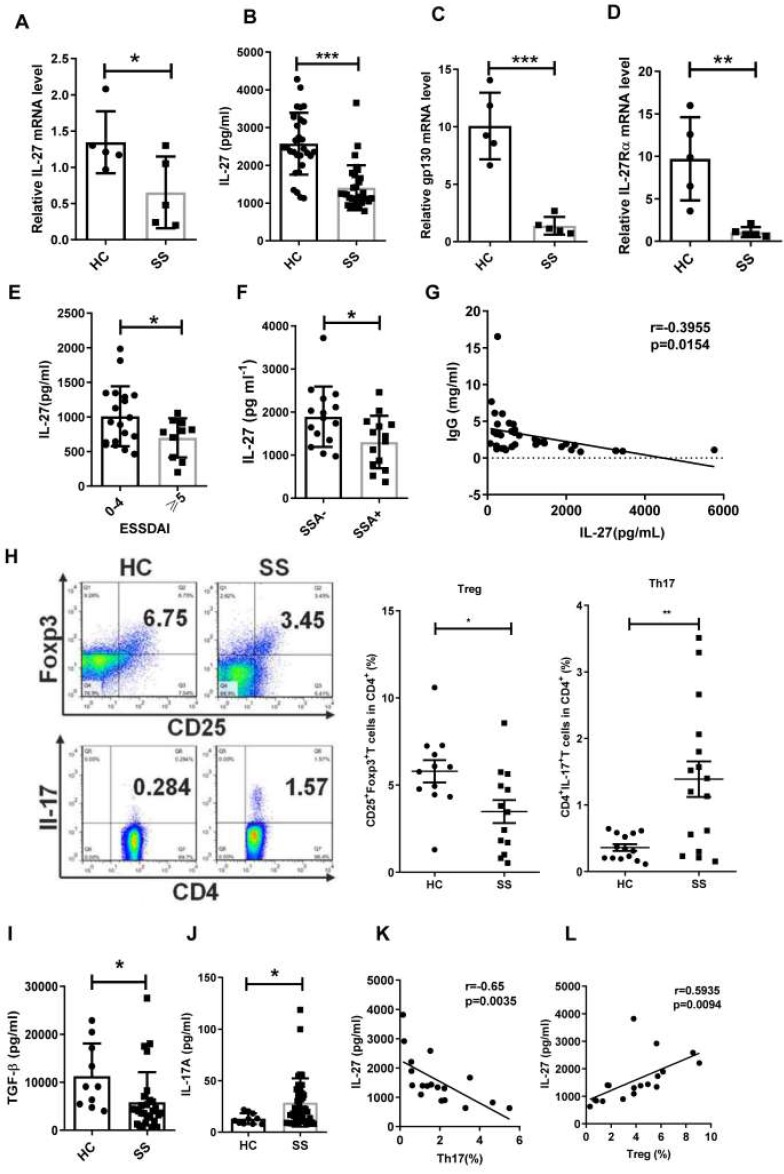
Serum IL-27 negatively correlates with disease activity in patients with Sjögren's syndrome. (**A, B**) IL-27 mRNA in PBMCs (**A**) and serum IL-27 (**B**) in patients with Sjögren's syndrome (SS) (n=30) compared with those of healthy controls (HC) (n=30). (**C,D**) Expression of IL-27 receptors, gp130 mRNA (**C**) and IL-27RαmRNA (**D**), were detected in PBMC from SS patients (n=5) and HC. (n=5) (**E**) Serum IL-27 was assessed according the European League Against Rheumatism (EULAR) Sjögren's syndrome Disease Activity Index (ESSDAI) scores. (**F**) Serum IL-27 was compared between SS patients with (n=14) and without anti-SSA antibody (n=15). (**G**) Correlation of serum IL-27 and IgG was analyzed. (**H**) Percentages of Th17 and Treg cells in SS patients (n=15) and HC (n=15) were shown. (**I, J**) Serum TGF-β (**i**) and IL-17A (**j**) in SS patients and HC were detected. (**K, L**) Correlations of IL-27 and Treg and Th17 cells were evaluated. Data were based on three independent experiments. Data are presented as mean±SEM. *, p<0.05, **, p<0.01, ***, p<0.001.

Although IL-12 family members IL-12, IL-23, IL-27 and IL-35, serve as important players in association with autoimmune diseases 26,27, their roles in SS remain largely unknown. To determine whether IL-12 family members are involved in the pathogenesis of SS, we measured the gene expression of IL-12, IL-23, IL-27 and IL-35 in PBMCs from SS patients and healthy controls by real-time PCR. We observed that the levels of IL-27 mRNA in SS PBMCs were significantly decreased when compared to healthy controls (**Fig. 1A**). IL-12 mRNA showed a significant increase in SS patients, while IL-23 and IL-35 mRNA remained unchanged compared to controls (**Supplementary Fig. 1**). In line with the reduction of IL-27 mRNA in PBMCs, lower levels of serum IL-27 were also detected in SS patients (SS 1407±109 pg/mL *vs* HC 2573±149 pg/mL) (**Fig. 1B**). The two subunits of IL-27 receptors, IL-27Rα and gp130, also exhibited a significant reduction in SS PBMCs (**Fig. 1C, D**).

To determine the clinical significance of IL-27, we assessed the correlation between IL-27 and European League Against Rheumatism (EULAR) Sjögren's syndrome Disease Activity Index (ESSDAI) scores. However, non-significant correlation existed between IL-27 and ESSDAI. We divided patients into two groups according to ESSDAI scores (0-4, inactive patients, ≧5, active patients). We found that IL-27 in inactive SS patients (2021±198 pg/mL) was higher than that in active SS patients (1395±162 pg/mL), indicating that IL-27 reflected the disease severity of SS patients (**Fig. 1E**). To determine the relationship of IL-27 and autoimmune antibodies in SS patients, we subgrouped the patients according to the anti-SSA or anti-SSB antibodies. IL-27 was significantly decreased in patients with positive anti-SSA (1304±163 pg/mL) compared to those patients with negative anti-SSA (1866±171 pg/mL) (**Fig. 1F**). The decreased IL-27 was also seen in patients with anti-SSB positive compared with patients with anti-SSB negative (**Supplementary Fig.2**). Since hypergammaglobulinemia is one of the immunological abnormalities in patients with SS, the relationship among IL-27 and IgG, IgM, IgA was also evaluated. The results showed that serum IL-27 negatively correlated with IgG in patients with SS (**Fig. 1G**), while serum IL-27 level showed no significant correlation with IgM and IgA (**Supplementary Fig.3**). These findings indicate that IL-27 is decreased and negatively correlated with disease activity in SS patients.

Since Th17 and Treg cells have been reported to play important roles in SS, we next determined the relationship between IL-27 and the Th17/Treg balance in SS patients. We observed that the frequency of Treg cells was decreased, while the frequency of Th17 cells was increased in SS patients compared to healthy controls (**Fig. 1H**). The ratio of Th17/Treg cells was significantly increased in SS patients. The change of Th17/Treg balance was correlated to the upregulation of IL-17A (HC 13.09±1.67 pg/mL *vs* SS 28.72±3.61 pg/mL) and downregulation of TGF-β in serum of SS patients (HC 11272±2162 pg/mL *vs* SS 5842±1162 pg/mL) (**Fig. 1, I, J**). Intriguingly, the frequency of Treg cells positively correlated with the level of IL-27, while the frequency of Th17 cells tend to negatively correlate with serum IL-27 level in SS patients (**Fig. 1 K, L**). These findings suggest that IL-27 may regulate Th17/Treg balance, and this regulation is disrupted in SS patients.

### IL-27 deficiency exacerbates SS-like symptoms in NOD mice

**Figure 2:**
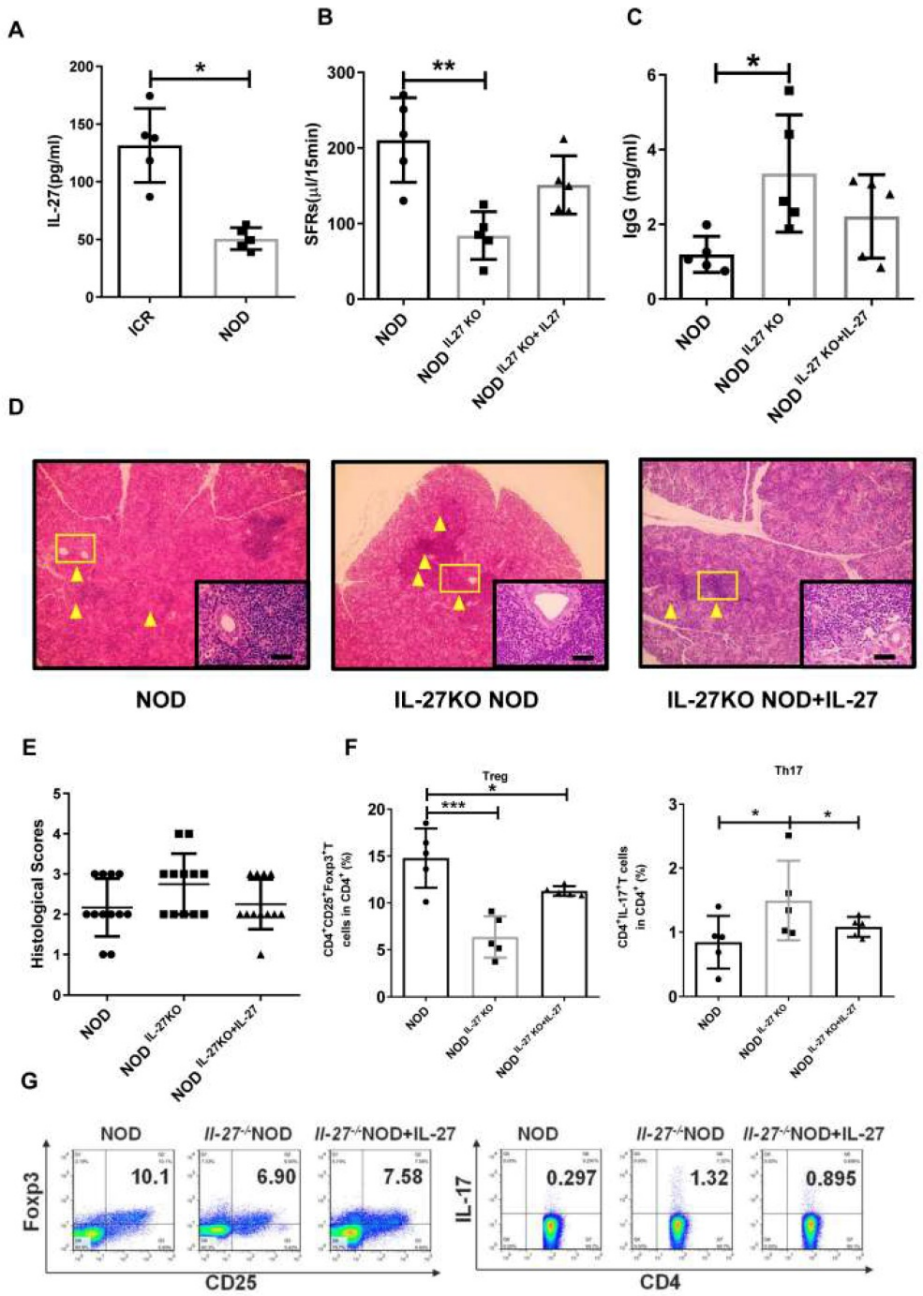
IL-27 deficiency exacerbates SS-like symptoms in NOD mice. (**A**) Serum IL-27 was compared between NOD (n=5) (12-week old) and control ICR mice (n=5) (12-week old). (**B**) Salivary flow rates were measured in wild type (WT) (n=5) (12-week old) and IL-27^-/-^ NOD mice(n=5) (12-week old). (**C**) Serum IgG in WT and IL-27^-/-^ NOD mice was compared (n=5) (12-week old). (**D**) Representative images of H&E staining of salivary glands in WT and IL-27^-/-^ NOD mice. Original magnification, ×40. Scale bars, 64 μm. Yellow arrows indicate infiltrating lymphocytes. (**E**) Lymphocyte infiltrations in salivary glands of mice were evaluated for histological scores. (**F and G**) Percentages of Treg and Th17 cells in WT (n=5) (12-week old) and IL-27^-/-^ NOD mice (n=5) (12-week old) were shown. Data were based on three independent experiments. Data are presented as mean±SEM. *,p<0.05, **, p<0.01.

Next, we examine the roles of IL-27 in NOD mice, a widely used SS model. We found that the level of IL-27 was significantly decreased in NOD mice (50.67±4.28 pg/mL) compared to control ICR mice (131.50±14.36 pg/mL) (**Fig. 2A**). The salivary flow rate (SFR) of NOD mice was remarkedly decreased than those of ICR mice (**Supplementary Fig. 4**). To determine whether IL-27 played a role in the SS-like symptoms, we generated IL-27 knockout NOD mice (IL-27^-/-^ NOD) (**Supplementary Fig. 5**) and found that the salivary gland function, assessed by the SFR, was compromised. IL-27^-/-^ NOD mice (84.0±14.2 μL/15min) exhibited significantly reduced SFR than wild-type NOD mice (WT NOD mice) (210.3±25.0 μL/15min) (**Fig. 2B**). IL-27^-/-^NOD mice produced approximately two-fold higher IgG than WT NOD mice, and this increase in IgG in IL-27^-/-^ mice was abolished with exogenous IL-27 treatment (**Fig. 2C**). However, the levels of IgM and IgA were compatible among all the mice (**Supplementary Fig.6**). Significantly increased lymphocytic infiltrates were observed in IL-27^-/-^ NOD mice compared to WT NOD mice (**Fig. 2D, E**). To further define the role of IL-27 in the pathogenesis of SS, we showed that administration of murine recombinant IL-27 into IL-27^-/-^ NOD mice ameliorated SS-like symptoms. One week after IL-27 injection, the SFRs of IL-27^-/-^ NOD mice were partially restored (**Fig. 2B**). Notably, histological analysis showed that IL-27 treatment significantly reduced lymphocytic infiltrates in IL-27^-/-^ NOD mice (**Fig. 2D, E**).

We next examined the levels of Th17 and Treg cells in IL-27^-/-^ NOD mice and found that the frequency and total number of splenic Th17 cells were increased compared to NOD mice (**Fig. 2F, G**). In contrast, the frequency and total number of splenic Treg cells were reduced in IL-27^-/-^ NOD mice (**Fig. 2F, G**). Interestingly, IL-27 treatment rescued the decreased number of Treg cells (**Fig. 2F, G**) and increased Th17 cells in IL-27^-/-^ NOD mice (**Fig. 2F, G**). These results highlighted that IL-27 deficiency exacerbated SS-like symptoms via increasing Th17 cells and decreasing Treg cells in NOD mice.

### MSC transplantation ameliorates SS-like symptoms by up-regulating IL-27

**Figure 3:**
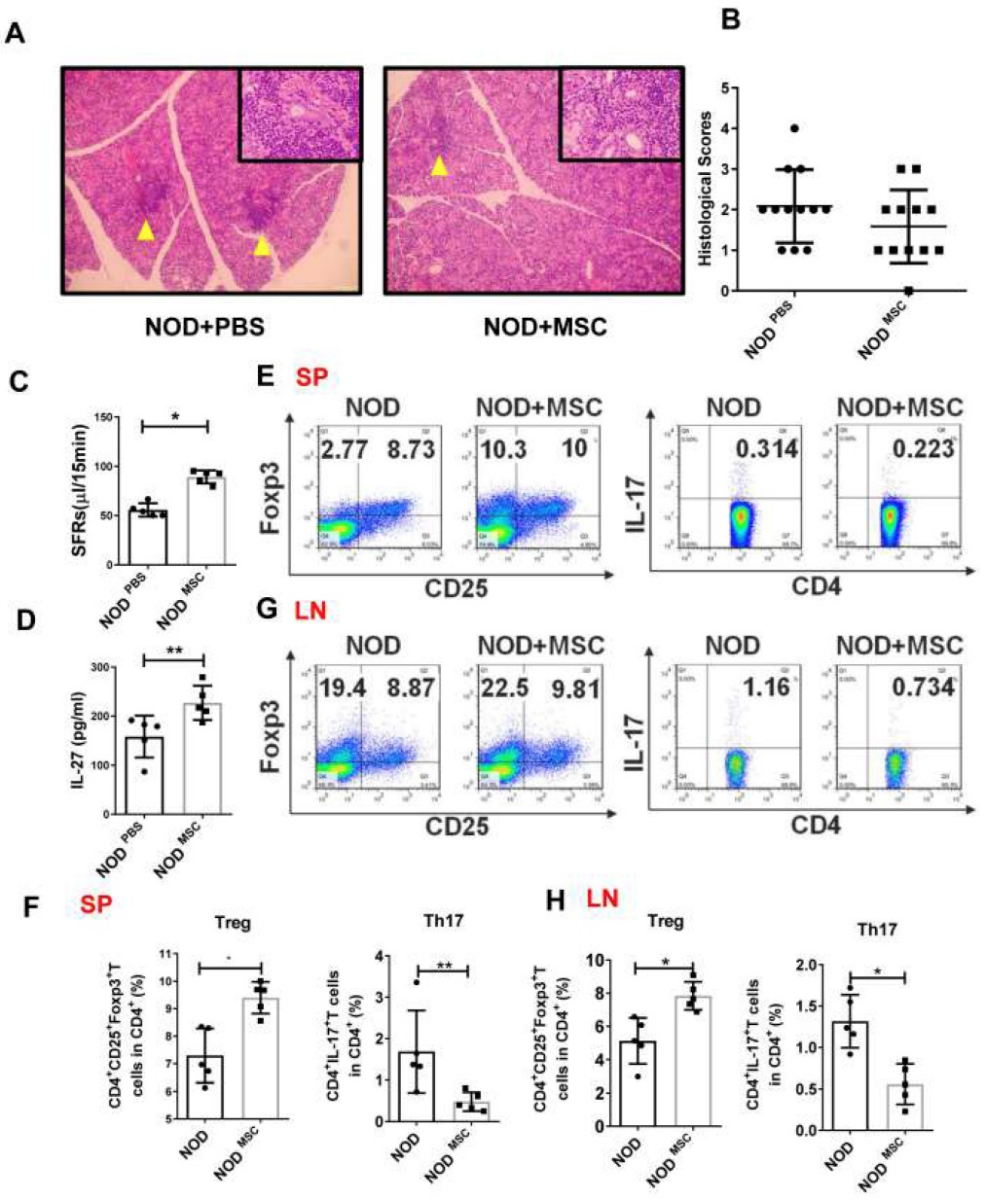
Mesenchymal stem cell (MSC) transplantation attenuates SS-like diseases in NOD mice. (**A**) Representative images of H&E staining of salivary glands in PBS (**A**) and MSC treated NOD mice. Yellow arrows indicate infiltrating lymphocytes. (**B**) Lymphocyte infiltrations in salivary glands of mice were evaluated for histological scores. (**C**) Salivary flow rates in PBS (n=5) (12-week old) and MSC treatment NOD mice (n=5) (12-week old). (**D**)Serum IL-27 was compared between PBS (n=5) (12-week old) and MSC treatment groups (n=5) (12-week old). (**E-H**) Representative FACS analysis of Treg and Th17 cells of spleens (**E, F**) and cervical lymph nodes (**G, H**)(n=5). Data were based on three independent experiments. Data are presented as mean±SEM. *,p<0.05, **, p<0.01.

To determine whether MSC transplantation has beneficial therapeutic effects in SS, we injected MSCs (1×10^6^ cells/mouse) into the tail vein in NOD mice and found that there was less inflammatory infiltration in the submandibular glands of MSC transplantation mice (**Fig. 3A, B**). The SFRs in MSC-treated mice (227.1±15.7 μL/15min) were significantly improved compared to PBS-treated mice (158.2±19.1 μL/15min) (**Fig. 3C**). We next investigated whether the effects of MSC transplantation on NOD mice were attributable to IL-27. First, we showed that serum IL-27 level in MSC-treated NOD mice (89.24±2.95 pg/mL) was increased compared to PBS-treated mice (55.63±3.02 pg/mL) (**Fig. 3D**). We then found that Th17 cells were markedly reduced and Treg cells were significantly increased in the spleens and cervical lymph nodes in MSC-treated mice (**Fig. 3E-H**). Altogether, these data indicate that MSC transplantation alleviates SS-like symptoms in NOD mice and the underlying mechanisms may relate to promoting IL-27 production, which modulates Th17/Treg balance.

### IL-27 regulates the differentiation of Th17 and Treg cells

**Figure 4:**
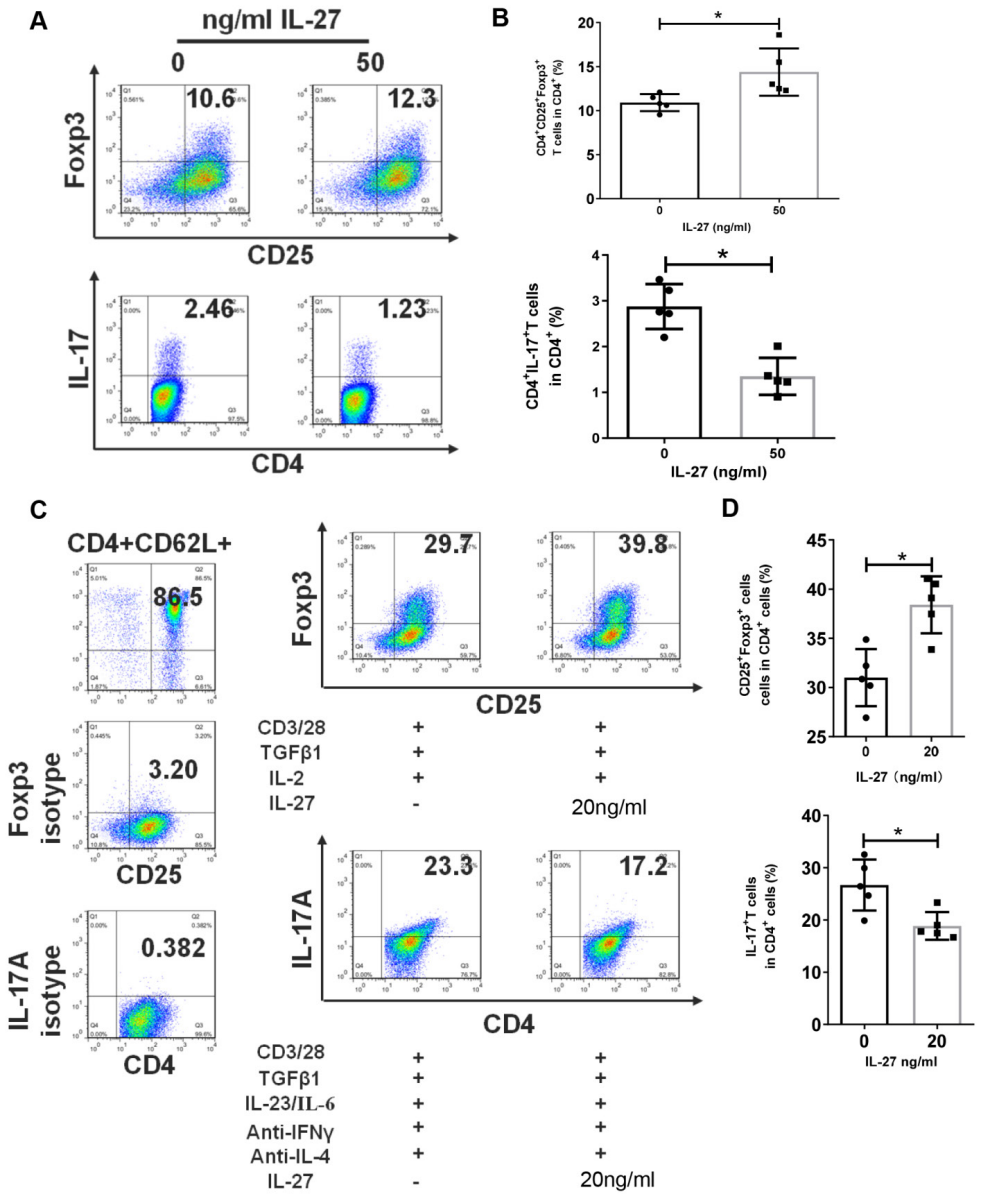
IL-27 modulates the proliferation and differentiation of Th17 and Treg cells in PBMCs and naïve CD4^+^ T cells. (**A, B**) Treg and Th17 cells in human PBMCs with and without IL-27 treatment. **(A)** Fluorescence staining of CD4, Foxp-3 and IL-17 in human PBMCs. (**B**) Summary of the frequencies of Treg and Th17 cells (n=5). (**C, D**) Flow cytometric analysis of Treg and Th17 cells, which were differentiated from mouse naïve CD4^+^ T cells in the presence or absence of IL-27 (n=5). Data are presented as mean±SEM. Data were based on three independent experiments. *, p<0.05.

The disruption of Th17/Treg balance and abnormal levels of IL-27 in SS patients and mice encouraged us to explore the regulation effects of IL-27 on Th17 and Treg cells. Firstly, we cultured PBMCs from SS patients with or without IL-27 and showed that IL-27 treatment increased the numbers of Treg cells, but suppressed Th17 cells (**Fig. 4A, B**). We next investigated whether IL-27 influenced the differentiation of Th17 and Treg cells. Naïve CD4+ T cells were purified from SS patients and then cultured to differentiate into Th17 or Treg cells in the absence and presence of recombinant IL-27. We found that IL-27 inhibited the differentiation of Th17 cells, but promoted Treg cell generation from naïve CD4+ T cells (**Fig. 4C, D**). These data suggest that IL-27 suppressed Th17 cells and increased Treg cells in SS patients.

### MSC transplantation increases the level of IL-27 in SS patients

**Figure 5:**
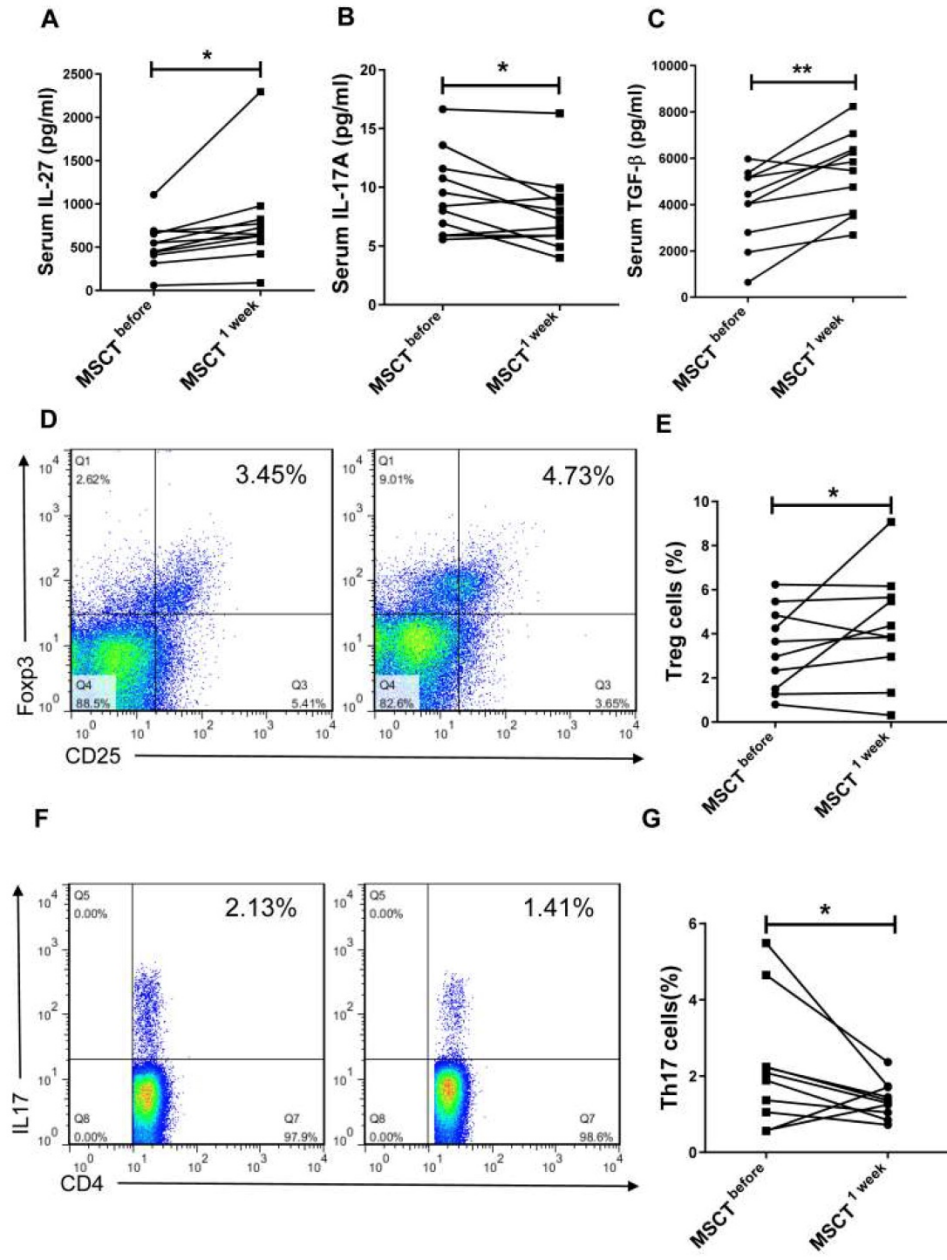
MSCs increase serum IL-27 and restore Th17/Treg balance in SS patients. (**A, B, C**) Serum IL-27 (**A**), IL-17A (**B**) and TGF-β (**C**) were determined in SS patients before and 1 week after MSC transplantation (n= 11). (**D, E, F**) Fluorescence staining of CD4, Foxp-3 and IL-17 in human PBMCs (**D, F**). Summary of Treg and Th17 cell frequencies in SS patients with MSC transplantation (**E, G**) (n=10). Data are presented as mean±SEM. Data were based on three independent experiments. *, p<0.05, **, p<0.01.

We next investigated whether MSC transplantation affected serum level of IL-27 and Th17/Treg cells in SS patients. We observed that MSC transplantation elevated the level of IL-27 (MSC 779.3±166.6 pg/mL *vs* PBS 534.8±79.27 pg/mL) (**Fig. 5A**) and reduced the level of IL-17A (MSC 7.88±1.01 pg/mL *vs* PBS 9.34±1.07 pg/mL) (**Fig. 5B**) in the blood of SS patients. Interestingly, the circulating level of TGF-β was also increased in SS patients after MSC transplantation (MSC 5223±522 pg/mL *vs* PBS 385±494 pg/mL) (**Fig. 5C**). Consistent with the reduction of IL-17A, the percentage of Th17 cells was also decreased following MSC transplantation, whereas the frequency of Treg cells increased (**Fig. 5D, E, F, G**). These results further support the notion that MSC transplantation promotes the production of IL-27, which in turn normalizes Th17/Treg cell balance in SS patients.

### MSCs trigger IL-27 production by IFN-β

**Figure 6:**
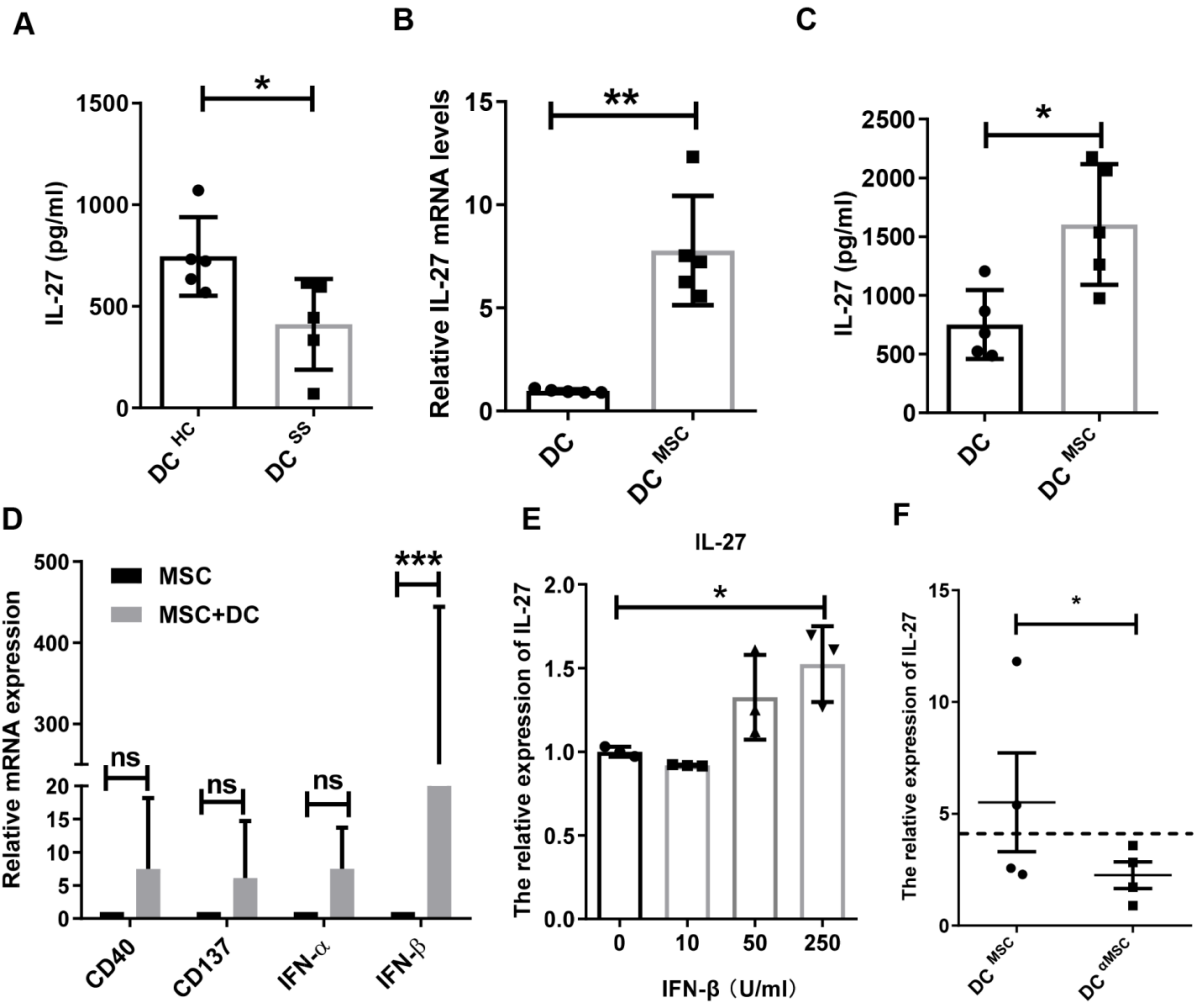
MSCs promote IL-27 production of dendritic cells through secreting IFN-β. (**A**) IL-27 produced by monocyte-derived DCs from SS patients (n=5) and HC (n=5). (**B, C**) IL-27 mRNA (**B**) and protein (**C**) of monocyte-derived DCs from HC with or without MSC treatment in the presence of 100ng/mL LPS was evaluated (n=5). (**D**) Genes related to IL-27 expression of DCs were screened and compared in MSCs co-cultured with and without monocyte-derived DCs from HC (n=4) in the presence of 100ng/mL LPS. (**E**) IL-27 mRNA expressions of monocyte-derived DCs with different concentrations of IFN-β treatment in the presence of 100ng/mL LPS (n=3). (**F**) IL-27 mRNA expressions of monocyte-derived DCs from HC co-cultured with MSC with and without IFN-β mRNA interference in the presence of 100ng/mL LPS (n=4). αMSC means siRNA silencing of IFN-β in MSCs. Data are presented as mean±SEM. Data were based on three independent experiments. *, p<0.05, **, p<0.01, ***, p<0.001.

It has been shown that IL-27 is mainly produced by dendritic cells (DCs), macrophages and monocytes 28. Based on the decreased IL-27 in SS patients, we hypothesized that these innate immune cells in SS patients produced less IL-27. Indeed, we found that monocyte-derived DCs in SS patients produced significantly less amount of IL-27 than that from control subjects (**Fig. 6A**), while macrophages and monocytes from SS patients produces similar levels of IL-27 compared to those from control subjects. To study MSC transplantation induces IL-27 in DCs, we cocultured MSCs with DCs and showed that MSCs indeed stimulated DCs to produce elevated levels of IL-27 mRNA and protein (**Fig. 6B, C**).However, MSCs failed to increase the IL-27 levels in the supernatants of cocultured macrophages and monocytes** (Supplementary Fig.7)**. We next investigated the mechanisms by which MSCs stimulate DCs to produce IL-27. As reported previously that a range of molecules, including CD 40, CD137, IFN-α/β, IFN-γ, and Toll like receptors were able to promote the expression of IL-27^29^, we measured the levels of CD40, CD137, IFN-α/β and IFN-γ in the MSCs cocultured with DCs. We found that IFN-β from the co-cultured MSCs were much higher and it was positively correlated with the increased levels of IL-27 from DCs (**Fig. 6D**), suggesting a role of IFN-β in the cocultures for IL-27 production in DCs. Indeed, we confirmed that exogenous IFN-β promoted the IL-27 production in DCs (**Fig. 6E**). Importantly, knockdown of IFN-β with specific siRNAs in MSCs substantially reduced IL-27 expression in DCs when co-cultured with MSCs (**Fig. 6F**). Taken together, these data suggest that IFN-β is required for MSC-mediated increase IL-27 production in DCs.

## Discussion

The molecular pathways responsible for the beneficial effects of MSC transplantation in SS patients remain incompletely understood. Here, we reported that MSCs ameliorated experimental Sjögren's syndrome via IFN-β dependent IL-27 in DCs. IL-27 in turn may regulate Th17/Treg balance. Supporting this conclusion include several lines of experimental evidence. First, deletion of IL-27 resulted in deteriorating SS-like symptoms and dysregulated Th17/Treg cell balance in NOD mice. Similarly, serum IL-27 was also decreased and Th17/Treg balance disrupted in SS patients. Second, MSC transplantation improved SS-like symptoms with increased IL-27 in NOD mice. The decreased IL-27 and Treg cells were partially restored in SS patients after MSC transplantation. Importantly, MSCs promote DCs to produce IL-27 in IFN-β dependent manner.

IL-27 has been reported to have pro- and anti-inflammatory activities in a variety of autoimmune diseases 18. For example, IL-27 is found to be elevated in RA patients and animal models compared with controls. However, in patients with SLE, reduced or increased IL-27 levels were reported in different studies, respectively 15. Here, we revealed a significantly reduction of serum IL-27 in patients with SS and NOD mice with SS-like disease. Previous studies have showed that IL-27 over-expression by rAAV-IL-27 vector in C57BL/6.NOD-Aec1Aec2 mice increased saliva secretion and decreased ANA formation 30. We found here that SS-like symptoms were aggravated in IL-27 knockout NOD mice. Moreover, we found that administration of IL-27 led to increased saliva secretion and reduced lymphocyte infiltrates in salivary glands in WT NOD mice (data not shown). However, Xia reported that serum IL-27 was markedly elevated in patients with SS and was particularly associated with interstitial lung disease 31. Although the reasons for these different observations remain to be unknown, it is likely due to the SS patients with interstitial lung disease in their study. As endothelial cells can also secrete IL-27 29, IL-27 production by cells of respiratory epithelium may be triggered by infiltrated lymphocytes in lung, which in turn resulted in increase of IL-27 in serum.

In patients with SS and mice with SS-like disease, Th17 cells have been detected within the lymphocytic foci in salivary glands 32. The percentage of Treg cells was reported to negatively correlate with clinical symptoms of SS patients 33,34. However, the available data only indicated the involvement of Th17 and Treg cells in the SS progression, the mechanisms related to Th17/Treg shift remained unknown. IL-27 has diverse influences on the regulation of T cell differentiation and immune response. IL-27 was able to regulate function of Th1, Th2, Th17, Treg, B cells and dendritic cells 35,36. Here we reported that IL-27 limited the severity of experimental SS by suppressing Th17 cell differentiation and promoting Treg cell differentiation. These findings may have implication for consideration of IL-27 as potential target for SS treatment, as elevated IL-27 might drive naïve T cells to differentiation into Treg cells rather into pro-inflammatory Th17 cells.

Hematopoietic stem cell transplantation (HSCT) has been used to treat severe autoimmune diseases. The rationale for autologous HSCT in autoimmune diseases was ascribed to abolish and reset the autoreactive immune system. However, the toxicity of HSCT limits the use of it on several autoimmune diseases. As far as we know, few patients with SS have been reported to receive HSCT and had unfavorable outcome 37,38. MSC therapy has been regarded as an encouraging approach for future treatment of autoimmune diseases, including SS 11,12,39. The MSCs for clinical use should be absence of contamination by pathogens, hepatitis B virus, hepatitis C virus, human immunodeficiency virus. The MSCs with cell viability greater than 90 %, have spindle-shaped morphology, characterization of CD73, CD105, CD90, and CD29 (>90%), and have no expression of CD45, CD34, CD14, CD79, and HLA-DR (<2%). *In vitro* osteogenic and adipogenic induction of MSCs was also demonstrated. Our previous study showed that no significant difference of therapeutic effects on SLE between single and double MSC transplantation 40,41. Both MSC and combined use of MSC plus complete Freund's adjuvant were effective in preventing saliva loss and reducing lymphocytic infiltration in salivary glands in NOD mice 42. We have previously demonstrated that MSC transplantation prevented and suppressed experimental SS-like diseases in NOD mice, and alleviate diseases in patients with SS 12. Recent study reported that MSCs had a therapeutic effect in NOD mice with relatively late stage SS 43. Here, we found that MSC treatment directed T cells toward Treg cells, while suppressed Th17 responses in patients with SS and NOD mice.

Significantly, we revealed a molecular mechanism underlying enhanced serum IL-27 and reestablishment of Th17/Treg cell balance in SS patients and mice after MSC transplantation. We showed that monocyte-derived DCs increased IL-27 expression after MSC treatment. Among the reported molecules that are involved in promoting production of IL-27 in DCs 29, we identified that IFN-β derived from MSCs represents a key factor to trigger monocyte-derived DCs production of IL-27. Supporting this conclusion include that IFN-β-silenced MSCs failed to promote IL-27 production in DCs. Cumulative evidences indicated that IFN-β had diverse biological activities, including antiviral, antimicrobial and immunomodulatory function 44. However, the fundamental role of IFN-β in the pathogenesis of SS is not well established. According to study of Bodewes, systemic type I IFNs were upregulated in 57% of the SS patients. Low IFN or negative of IFN were in subsets of these patients 45. Several new approaches and strategies to downregulate the type I IFN have showed beneficial effects on SS, with improvement demonstrated in exocrine histopathology as well as dryness symptoms and secretory function 46. However, the results of Benchabane's group suggested that IFN-β restored immune balance between inflammatory and anti-inflammatory responses by reducing pro-inflammatory cytokines in peripheral blood mononuclear cells from SS patients. Therefore, it seemed that IFN-β had beneficial impact in SS 47. Therefore, further studies are needed to uncover the exact role and precise mechanisms of IFN-β in SS.

In conclusion, this study identifies a role of DC-derived IL-27 in suppressing the pathogenesis of Sjögren's syndrome. We have revealed a novel mechanism by which MSCs induce IL-27 production by DCs through IFN-β and consequential the restoration of the normal Th17/Treg balance.

## Supplementary Material

Supplementary figures and tables.

## Abbreviations

MSC: mesenchymal stem cell
SS: Sjögren's syndrome
IL-27: interleukin 27
NOD: non-obese diabetic
DCs: dendritic cells
UC-MSCs: umbilical cord-derived MSCs
PBMCs: peripheral blood mononuclear cells
ESSDAI: European League Against Rheumatism (EULAR) Sjögren's syndrome Disease Activity Index
IFN-β: interferon-β
Treg cells: regulatory T cells
Th17 cells: T helper 17 cells
IL-27R: IL-27 receptors
ICR: Institute of Cancer Research
SFR: salivary flow rate
HSCT: Hematopoietic stem cell transplantation
