# Supplementary material for: Mesenchymal stem cell transplantation alleviates experimental Sjögren's syndrome through IFN-β/IL-27 signaling axis
Source: Theranostics. 2019 Oct 21;9(26):8253–65. doi: 10.7150/thno.37351 (PMC6857067; doi:10.7150/thno.37351)
Supplement: Supplementary file 1 — Supplementary figures and tables. [file thnov09p8253s1.pdf]

Supplemental materials

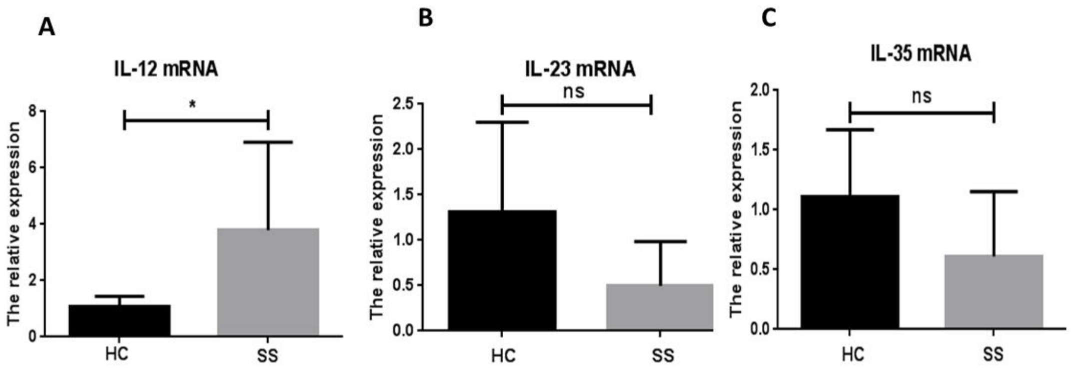

Figure S1 IL-12, 23 and 35 mRNA expressions in PBMCs. (A) IL-12 mRNA was elevated in SS patients, compared with healthy controls. (B and C) IL-23 and 35 mRNA expression was comparable between SS patients and healthy controls.

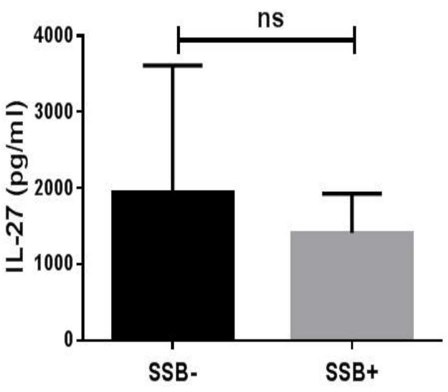

Figure S2 IL-27 correlated with autoantibodies in patients with SS. Serum IL-27 tended to reduce in SS patients with anti-SSB antibody.

A

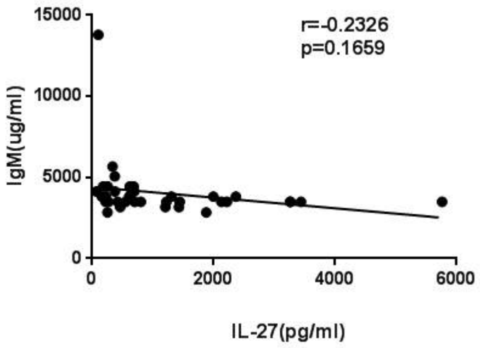

B

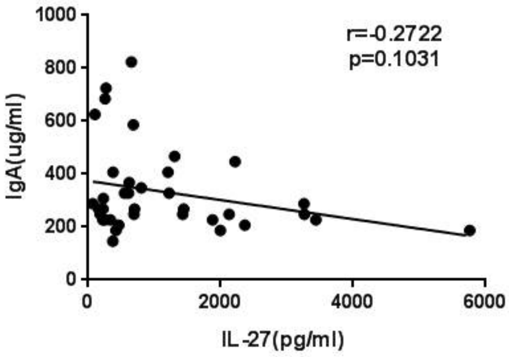

Figure S3 IL-27 correlated with immunoglobulin M and A in patients with SS. (A and B) IL-27 showed a negative correlation with IgM and IgA in SS patients.

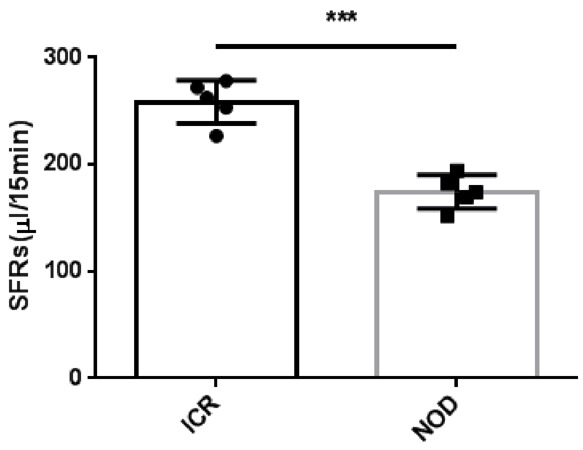

Figure S4 Salivary flow rates were measured in ICR and NOD mice (n=5).

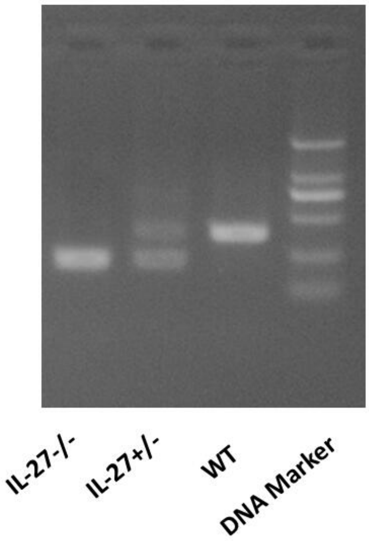

Figure S5 The genotype of IL-27-/- NOD mice. The genotype was determined by PCR.

A

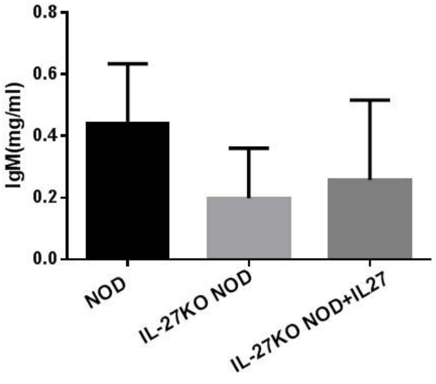

B

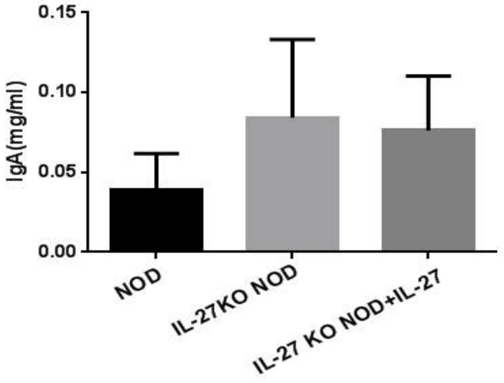

Figure S6 IgM and IgA in IL-27 KO mice. (A and B) IgM and IgA were comparable among WT, IL-27 KO, IL-27 KO with recombinant IL-27 treatment NOD mice.

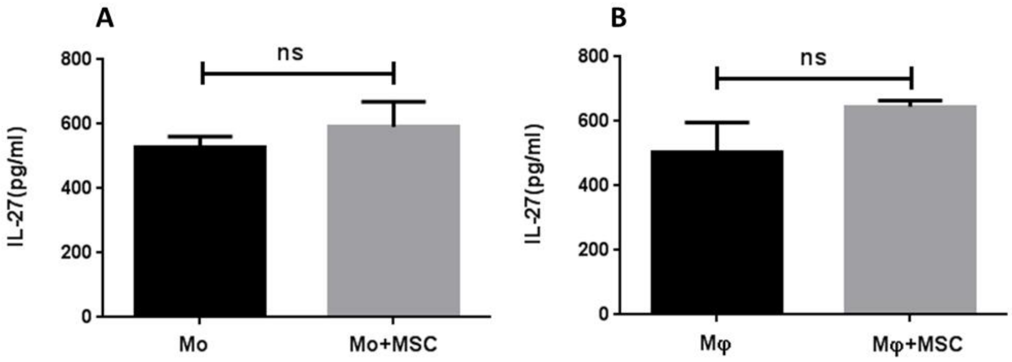

Figure S7 Mesenchymal stem cells promote the production of IL-27 by macrophages and monocytes. (A and B) Supernatants of macrophages

and monocytes with MSC treatment showed a tendency of increased IL-27 levels.

Supplementary Table 1 Baseline clinical characteristics and medications for the SS patients

Patient	Age/sex	Disease duration (months)	ESSDAI	Medication

1	49/F	12	4	Pred,HCQ,CYC
2	22/F	72	3	Pred,HCQ,CYC
3	53/F	24	3	Pred,HCQ,LEF
4	51/F	9	5	Pred,HCQ, CYC
5	50/F	24	6	Pred,HCQ,CYC,LEF
6	26/F	1	5	No medication
7	73/F	36	5	Pred,HCQ,CYC
8	59/F	60	5	Pred,HCQ,cyclosporin
9	61/M	24	1	Pred,HCQ,LEF
10	52/F	84	9	Pred,HCQ,Tacrolimus
11	46/F	24	6	Pred,HCQ,CYC,cyclosporin
12	55/F	1	8	No medication
13	52/F	60	4	Pred
14	53/F	240	13	Pred,HCQ,CYC,LEF
15	70/F	24	3	Pred,HCQ,
16	53/M	120	5	Pred,HCQ,
17	43/F	2	5	Pred
18	36/M	6	7	Pred,HCQ,CYC, Triptolide
19	63/F	36	2	Pred,HCQ,
20	34/F	120	4	Pred
21	54/F	1	1	No medication
22	34/F	120	6	Pred,HCQ
23	60/F	72	5	Pred,HCQ,LEF
24	20/F	12	8	Pred,HCQ,CYC,LEF

25	55/F	84	8	Pred,HCQ,CYC,LEF
26	44/F	6	4	Pred,HCQ, Triptolide
27	57/M	24	7	Pred,HCQ,CYC, cyclosporin
28	64/M	120	7	Pred,HCQ, CYC
29	29/F	72	6	Pred,HCQ,LEF
30	53/F	96	4	Pred,HCQ,CYC
31	60/F	240	8	Pred,HCQ
32	32/F	3	10	Pred,HCQ, CYC,LEF, Tacrolimus
33	39/F	72	8	Pred,HCQ,CYC
34	73/F	240	6	Pred
35	52/F	60	7	Pred,HCQ, Tacrolimus
36	46/M	36	3	Pred,HCQ, CYC
37	49/F	6	11	Pred,HCQ,LEF
38	45/F	12	5	Pred,HCQ, CYC

Pred, prednisone; HCQ, hydroxychloroquine; CYC, cyclophosphamine; LEF, leflunomide;

Supplementary Table 2. Primers for real-time PCR

Gene(human)		Primers	Product length(bp)
IL-27	Forward	5’-TGCCAGGAGTGAACCTGTACC -3’	
	Reverse	5’-CGTGGTGGAGATGAAGCAGA -3’	119
IL-12	Forward	5’-AGGAATGTTCCCATGCCTTCA-3’	
	Reverse	5’-CCAATGGTAAACAGGCCTCCAC-3’	170

IL-23	Forward	5’-GAACAACTGAGGGAACCAAACC -3’	
	Reverse	5’-GAATCTCTGCCCACTTCCACTT -3’	80
IL-35	Forward	5’-GACCTCACAGACTACGGGGAAC -3’	
	Reverse	5’-CGGGAAGCCCTTGCTACTT -3’	85
WSX-1	Forward	5’-CCACCTACATGGCCATCTCATAAA-3’	
	Reverse	5’-GCCAGTGGCTAGCTCCAAATC-3’	147
gp-130	Forward	5’-CTGGGAGTGCTGTTCTGCTTTAATA-3’	
	Reverse	5’-TGAGGTGACCACTGGGCAATA-3’	104
GAPDH	Forward	5’-GCACCGTCAAGGCTGAGAAC-3’	
	Reverse	5’-TGGTGAAGACGCCAGTGGA-3’	100
